# Impact of Autoclaving on the Dimensional Stability of 3D‐Printed Guides for Orthodontic Mini‐Implant Insertion – An In Vitro Study

**DOI:** 10.1002/cre2.70111

**Published:** 2025-03-07

**Authors:** Samuel David, Mira Hüfner, Nicole Rauch, Robert Kerberger, Dieter Drescher, Giulia Brunello, Kathrin Becker

**Affiliations:** ^1^ Department of Orthodontics University Hospital of Düsseldorf Düsseldorf Germany; ^2^ Department of Oral Surgery University of Düsseldorf Düsseldorf Germany; ^3^ Department of Orthodontics and Dentofacial Orthopaedics Charité ‐ Universitätsmedizin, Freie Universität Berlin and Humboldt‐Universität zu Berlin Berlin Germany; ^4^ Department of Neurosciences, School of Dentistry University of Padua Padua Italy

**Keywords:** 3D printing, insertion guide, micro‐CT, optical 3D scanner, sterilization

## Abstract

**Objectives:**

This study aimed to assess the effect of the printing process itself and steam autoclaving on the geometrical stability of 3D‐printed guides for mini‐implant insertion.

**Material and Methods:**

Fifty guides (*n* = 10 per group) were printed with five printer/resin combinations (PRCs) from the same STL file using either digital light processing (DLP/EG, DLP/Next, DLP/Opti), desktop stereolithography (SLA/DSG) or liquid crystal display stereolithography printers (LCD/Amber). Half were sterilized by steam autoclaving with Cycle 1 (121°C, 1 bar, 20.5 min), half with Cycle 2 (134°C, 2 bars, 5.5 min). Before (T0) and after sterilization (T1) the guides were scanned with a structured light 3D scanner, and selected guides also with micro‐CT for validation. Linear measurements were performed in three axes on STL, and on T0 and T1 scans. Linear mixed‐effects models were used, followed by post‐hoc tests in case of significance.

**Results:**

Measurements at T0 and T1 differed significantly from STL in both *x*‐ and *y*‐axis (4 and 3 PRCs, respectively) (*p* < 0.05); in *z*‐axis only DLP/Next showed significant differences between T0 and STL (*p* < 0.001). The comparison between T0 and T1 revealed significant differences in *x*‐axis for DLP/Next and DLP/Opti after Cycle 1 and Cycle 2, respectively (*p* < 0.05), while in the *y*‐axis no intra‐group difference was recorded. In the *z*‐axis all PRCs except for SLA/DSG exhibited significant shrinkage (for Cycles 1 or 2). Differences between the two cycles at T1 were registered only in *z*‐axis (DLP/Next and LCD/Amber).

**Conclusions:**

Compared with the original, all PRCs except for SLA/DSG presented significant changes in their dimensional stability owing to the printing process itself and/or the sterilization. If these changes are of clinical significance, they remain to be verified.

**Clinical Relevance:**

With the utilized design, the guides fabricated with SLA provided lower dimensional changes as compared to the ones produced by the other printing techniques.

## 1 Introduction

Since their first conception in 1989 (Arthur and Berardo 1989), orthodontic mini‐implants have been successfully used for a variety of indications, including tooth intrusion, extrusion, or molar distalization, with a reduced need for patient compliance. Meanwhile, mini‐implants offer a form of anchorage that does not involve other teeth and does not lead to undesired movement of the anchorage unit (Leo et al. 2016; Li et al. 2020; Magkavali‐Trikka et al. 2018).

The correct position of orthodontic mini‐implants is of great importance and factors such as root proximity, bone support and soft tissue thickness contribute to the selection of an ideal implant site (Shinohara et al. 2013). Several studies have reported that a lack of accuracy during the insertion process of orthodontic mini‐implants could lead to the damage of adjacent teeth as well as to undesired implant loss (Mihit Mihit et al. 2023; Reynders et al. 2009). Even experienced clinicians could profit from computer‐guided implant placement (Vermeulen 2017), which also offers orthodontics the chance to place the mini‐implants and the orthodontic appliance, designed based on the digital planning, in a single session (Wilmes et al. 2019).

The evaluation of the insertion pathway is needed to minimize the risk of iatrogenic damages to the neighboring structures (Giudice et al. 2021; Inchingolo et al. 2023). Despite the currently available low‐dose scanning protocols of CBCT scans, it is preferable not to expose especially young patients to ionizing radiations, if not strictly necessary (Scarfe 2012). This has led to the search for a safe space in the palate for the insertion without the need for routine cone beam computed tomography (CBCT) examinations. The so‐called T‐Zone is assumed to be an ideal area for mini‐implant insertion in the anterior palate with sufficient available bone of adequate density and low risk for iatrogenic damage (Becker et al. 2019). The T‐Zone consists of a T‐shaped area in the anterior palate located posteriorly to the palatal rugae. This T‐Zone allows for the insertion of mini‐implants either in the median or paramedian area (Wilmes et al. 2016). Suitable sites for the insertion of mini‐implants were also identified about 2–5 mm from the alveolar crest in the palate between the second premolar and the first molar (Poggio et al. 2006), as well as in the area between the lateral incisor and the first premolar (Alsamak et al. 2013). Nonetheless, the correct placement and angulation of the mini‐implants is fundamental to guarantee the safety of the procedure. Recent studies have shown the advantages of using insertion guides for orthodontic mini‐implants in the anterior palate over free‐hand placement (Kniha et al. 2021). Guides are associated with an increase in accuracy in the mini‐implant insertion, lower failure rates, and increased implant stability (Jedliński et al. 2021). In addition, it has been reported that tooth‐borne guides are more precise compared to mucosa‐borne ones (Jedliński et al. 2021; Möhlhenrich et al. 2019). Studies have shown that the quality of intraoral scanner or cone beam computed tomography (CBCT) scanners could also have an effect on the precision of guided implant insertion (Gimenez et al. 2015; Tadinada et al. 2015).

Complications and difficulties can arise when using insertion guides, such as limited access and visibility due to the presence of the guide, or the fracture of the guide itself (Sicilia and Botticelli 2012). Guides are commonly produced by desktop 3D printers utilizing resin‐based materials (D'haese et al. 2017), but CAD/CAM milling is reported to offer similar levels of transfer accuracy (Schwärzler et al. 2024). This offers the possibility to fabricate precise insertion guides in a timely manner and at a relatively low cost (Deeb et al. 2017). The majority of the resins available for this purpose fulfills the necessary requirements to be used as guides for implant placement. Indeed, these guides qualify under US and EU law as Medical Devices (IA/critical A), and thus their sterilization is required before usage (Regulation [EU] 2017; Rutala et al. 2008). In a previous study of our group, autoclaving process seemed to have an effect on the mechanical properties of the 3D‐printed guides (Kirschner et al. 2022), and changes in the Vickers hardness were registered after autoclaving for several tested materials. Nevertheless, changes in flexural modulus were detected only for one material out of five tested, which presented a significant decrease in the flexural modulus only when using the highest temperature and pressure and not after autoclaving at 121°C and 1 bar for 20.5 min. However, the influence of autoclaving parameters on the dimensional stability of the insertion guides produced with those printer/resin combinations (PRCs) remains to be investigated.

We have previously assessed the impact of autoclaving on the dimensional stability of 3D‐printed guides for dental implant insertion in a study with the same design as herein (Hüfner et al. 2024). Thus, the present study may be considered somehow repetitive. Nevertheless, there are not only major differences between orthodontic and dental implants (e.g., dimensions, installation, location, and related template shape) but also implantology and orthodontics have a different readership, and orthodontists would most probably not consider a paper on surgical guides relevant; this, in turn justified the current study.

Therefore, the null hypothesis was that the printing process and the sterilization via autoclaving had no significant influence on the dimensional stability of 3D‐printed resin‐based mini‐implant insertion guides. Therefore, this study aimed to evaluate the influence of the printing process and of steam autoclaving parameters on the dimensional stability of the insertion guides, manufactured using different combinations of resin materials and printing methods.

## 2 Methods

The article is reported in compliance with the modified Consolidated Standards of Reporting Trials (CONSORT) guidelines for reporting in vitro studies on dental materials (Appendix 1) (Faggion 2012).

### 2.1 Design of an Insertion Guide for Orthodontic Mini‐Implants

**Figure 1:**
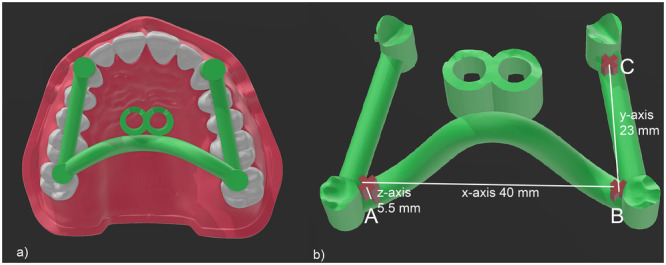
(a) Scanned maxillary model with insertion guide. (b) Computer‐aided design (CAD) model of the designed insertion guide. The three markers (A, B, C) and the distances for *x*‐, *y*‐, and *z*‐axis are indicated in white.

A maxillary model (Frasaco GmbH, Tattnang, Germany) was scanned with an intraoral scanner (3Shape TRIOS, Copenhagen, Denmark) and an insertion guide for orthodontic mini‐implants was designed on the resulting STL file using Blender (Stichting Blender Foundation, Amsterdam, Netherlands) (Figure 1). Three cross‐shaped markers (A, B, C) were added to the 3D model of the insertion guide using the software 3D Builder (Microsoft Corporation, Redmond, WA, USA) to enable the measurement of possible dimensional changes after autoclaving. The markers A, B, and C were designed so that the distances between them were the following: A–B = 40 mm, B–C = 23 mm, A height = 5.5 mm.

### 2.2 3D Printers and Resins

**Figure 2:**
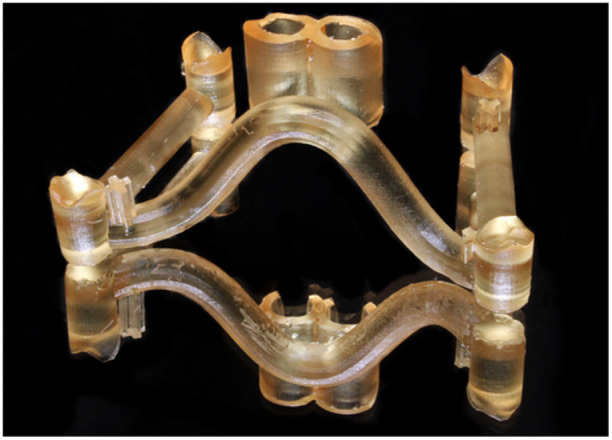
Representative image of a 3D‐printed guide.

50 skeletonized insertion guides for orthodontic mini‐implants were printed with five different PRCs (*n* = 10 per group) starting from the same guide design (Figure 2), as summarized in Table 1. Three different digital light processing (DLP) printers, one desktop stereolithography (SLA) printer, and one liquid crystal display stereolithography (LCD‐SLA) printer were utilized. All printer/resin combinations were printed at a layer height of 100 µm except for DLP/EG at a layer height of 50 µm. All investigated guides were printed parallel to the print bed with their intaglio surfaces facing away from the print bed. The respective manufacturers' post‐processing guidelines were followed for all materials. All guides were cleaned in a 99.9% isopropyl alcohol solution and subsequently post‐cured for 10 min.

**Table 1:** Details of the 3D printers, resins, and post‐processing methods used in the five groups.

Group acronym^a^	Printing method	3D Printer	Resin materials	Resin	Manufacturer(s)	Post‐processing
DLP/EG	DLP	Micro Plus XL	Acrylate/methylacrylated oligomers and monomers, photo initiators, colorants/dyes and absorbers	E‐Guide	Envisiontec Inc. Dearborn, MI, USA	Otoflash G171 (NK‐Optik) 2 × 1000 flashes once each side under inert nitrogen gas
DLP/Next	DLP	NextDent 5100	Monomer based on acrylic esters, ethoxylated bisphenol A, Methacrylic oligomer, Phosphine oxide	NextDent SG	Vertex‐Dental B.V., Soesteberg, Netherlands	RS cure (Rapidshape) 10 min
DLP/Opti	DLP	ASIGA MAX	Bifunctional methacrylate, phosphine oxide	Optiprint Guide	Pluradent GmbH & Co. KG, Offenbach, Germany (printer); dentona AG, Dortmund, Germany (resin)	Otoflash G171 (NK‐Optik) 2 × 2000 flashes once each side under inert nitrogen gas
SLA/DSG	SLA	Form 3	Ethoxylated bisphenol A dimethacrylate, 7,7,9(or 7,9,9)‐trimethyl‐4,13‐dioxo‐3,14‐dioxa‐5,12‐diazahexadecane‐1,16‐diyl bismethacrylate, phenyl bis(2,4,6‐trimethylbenzoyl)‐phosphine oxide	Dental SG	Formlabs Inc. Sommerville, MA, USA	Form Cure (Formlabs) 10 min at 60°C
LCD/Amber	LCD‐SLA	Slash Plus	Acrylate monomer, acrylate oligomer, photo initiator(s)	zSG Amber	UniZ Technology LLC., San Diego, CA, USA	U Cure (Uniz) both sides 1 min level 2

Abbreviations: DLP, digital light processing; LCD‐SLA, liquid crystal display stereolithography; SLA, stereolithography.

^a^ *n* = 10 per group.

All the resins but one (i.e., DLP/EG) were authorized by the manufacturers for steam autoclaving and printing with the printer used. DLP/EG was authorized by the manufacturer for immersion disinfection but not for steam autoclaving.

### 2.3 Sterilization Process

All templates were sterilized by vacuum steam autoclaving (Vacuklac 44‐B, MELAG oHG, Berlin, Germany). To assess the effect of autoclaving parameters (i.e. temperature pressure, and time), half of the templates (*n* = 5 per group) were sterilized at 121°C, 1 bar and 20.5 min (Cycle 1), whereas the other half at 134°C, 2 bar and 5.5 min (Cycle 2).

### 2.4 Structured Light 3D Scanning

Before (T0) and after (T1) autoclaving, all templates were scanned using a structured light 3D scanner (Freedom UHD, DOF Inc. Soul, South Korea) with an accuracy of 7 µm, using the triangulation method.

**Figure 3:**
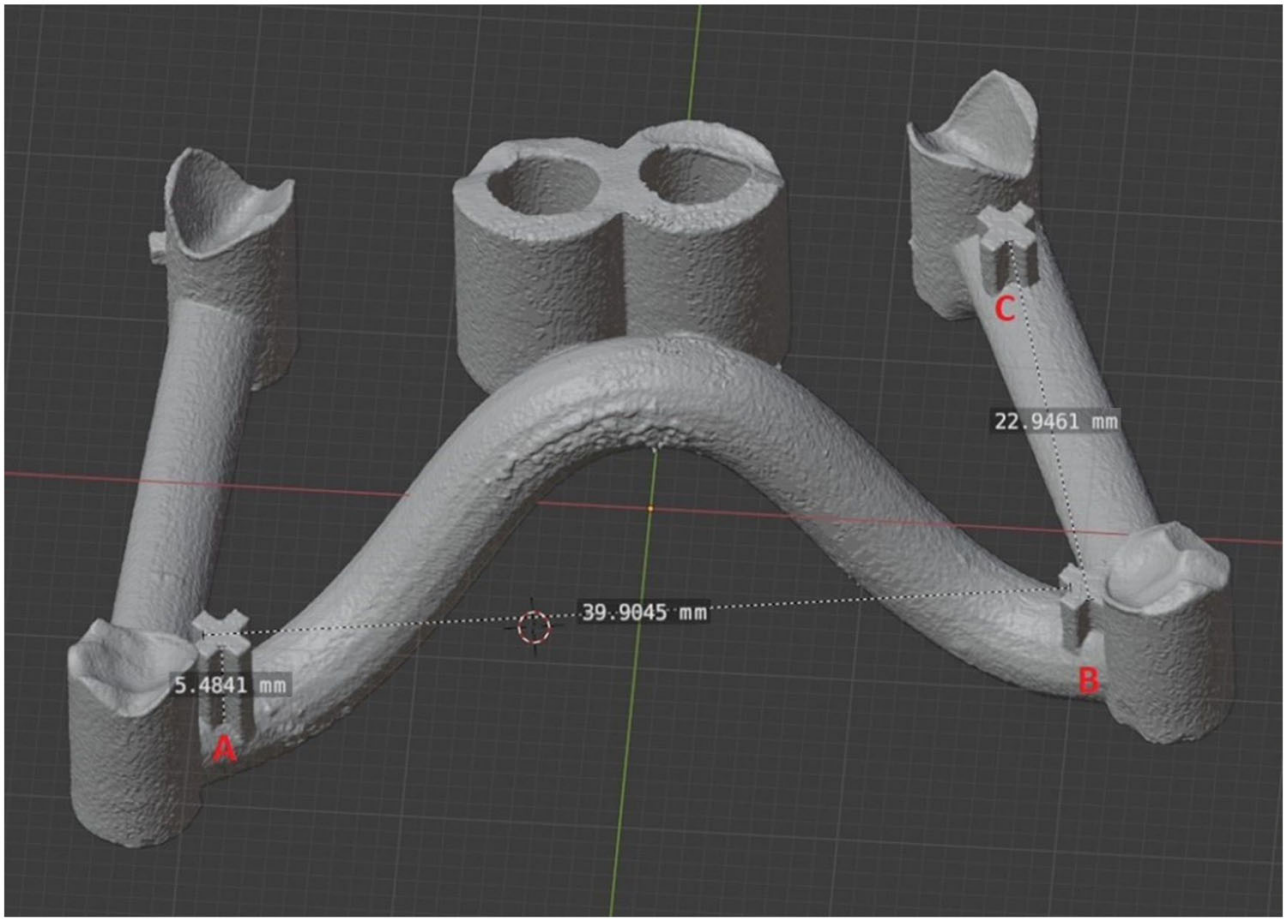
Representative image of measurement collection.

The markers (A, B, C) displayed in the 3D models were used as measuring points to determine changes in the 3D geometry by one blinded examiner (S.D.) using Blender (Blender Foundation, Amsterdam, Netherlands) (Blender Online Community 2018) (Figure 3).

Changes within the *x*‐axis were monitored by measuring the distance between the lateral concave vertex of the cross‐shaped marker A and the mesial concave vertex of the cross‐shaped marker B (both positioned in the molar zone, as shown in Figure 1). Regarding the *y*‐axis, changes were monitored by measuring the distance between the posterior concave vertex of the cross‐shaped markers B and the posterior concave vertex of the cross‐shaped marker C (positioned in close proximity to the canine stop, as shown in Figure 1). The height of marker A on the orthodontic mini‐implant templates was used to measure changes in the *z*‐axis. All the measurements were performed before (T0) and after (T1) autoclaving, as well as in the original STL files.

### 2.5 Microcomputed Tomography (Micro‐CT) and Image Processing

To validate the structured light 3D (SL) scanning measurements, 2 templates per group were scanned with a micro‐CT scanner (VivaCT 80, Scanco Medical AG, Brüttisellen, Switzerland) before and after autoclaving (T0 and T1, respectively). The scans were performed at 45 kVp, 88 μA, and 107 ms integration time and reconstructed to a nominal isotropic voxel size of 39 μm. Conversion to STL was performed using an internally programmed script based on Image Processing Language (IPL) (Scanco Medical AG, Brüttisellen, Switzerland). First, in the uct_evaluation software (Scanco Medical AG), the outer boundaries of the templates were roughly drawn in the form of a volume of interest (VOI) by two trained observers (R.K./N.R.). The script, then, segmented the templates within the VOI with a threshold of 4.2% and the segmented image was converted to an STL file.

The segmented guides were exported as STL files and evaluated in Blender (Blender Foundation) (Blender Online Community 2018). The same distances from A to B and B to C, as well as the height of A in *z*‐axis, were measured. These measurements were used to compare the relative distances for micro‐CT and their respective SL scan counterpart to assess the reliability of the SL scans, not to evaluate the accuracy of the 3D scanning itself.

### 2.6 Intrarater Reliability

All micro‐CT and SL scans were measured by one trained and blinded observer (S.D.). Measurements were performed on micro‐CT scans of 10 guides (two for each printer/resin combination), which were re‐measured after 2 and 3 weeks from the first assessment. The procedure was carried out for both T0 and T1 micro‐CT scans. The respective SL scans were re‐measured at 12 and 13 weeks after the first measurement.

### 2.7 Statistical Analysis

Data was analyzed using the open‐source program R (R Core Team 2022) and boxplots were created for descriptive purposes with the R package ggplot2 (Wickham 2016). A convenient sample size was selected based on previous publications on the topic (Bayarsaikhan et al. 2021; Pop et al. 2022). To assess the reliability of the SL scanning/manual measurements, intraclass correlation coefficients (ICC) were calculated for the repeated measurements. The Bland Altman analyses were performed for method comparison between SL and micro‐CT measurements (Datta 2017). The ICC was computed to assess intra‐ and interobserver agreement. For each of the five resin/printer combinations, the dimensional values (mean ± standard deviation) in the three axes of the printed guides before (T0) and after autoclaving (T1) were calculated. To assess if there were dimensional alterations per axis among the different time points (i.e., original STL, printed guides at T0 and T1), linear mixed‐effects analyses were carried out with the R package lme4 (Bates et al. 2015). The dimensional values of the original STL file, of the printed guides before (T0) and after autoclaving (T1 – Cycle 1, T1 – Cycle 2) were entered as fixed effects into the model. For random effects, intercepts for the guides were defined. Likelihood‐ratio tests were used to obtain P values by comparing the model with and without the effect in question. When significant, a Tukey post‐hoc test was conducted using the glht‐package (Hothorn et al. 2008). No obvious deviation from homoscedasticity or normality was revealed at visual inspection of residual plots. Statistical significance was set at a P value of 0.05.

## 3 Results

Details on the adherence to the modified CONSORT criteria (Faggion 2012) are shown in Appendix 2.

### 3.1 SL Scan Reliability

The correlation coefficient between measurements performed on micro‐CT and SL scans resulted to be 0.999 (95% CI: 0.999–1.0, *p* < 0.001) (ICC: 1, *p* < 0.001), thus confirming the high reliability of the measurements. In one SL scan of the LCD/Amber combination (at T0) the markers on the guide were not clearly detectable, even after numerous attempts. Therefore, this SL scan and related micro‐CT scan could not be considered for the analysis.

### 3.2 Intrarater Reliability

For repeated measurements of SL scans the correlation coefficient amounted to 1 (*p* < 0.001). Similarly, the correlation coefficient for repeated measurements performed on micro‐CT scans resulted equal to 1 (*p* < 0.001).

### 3.3 Dimensional Changes

**Figure 4:**
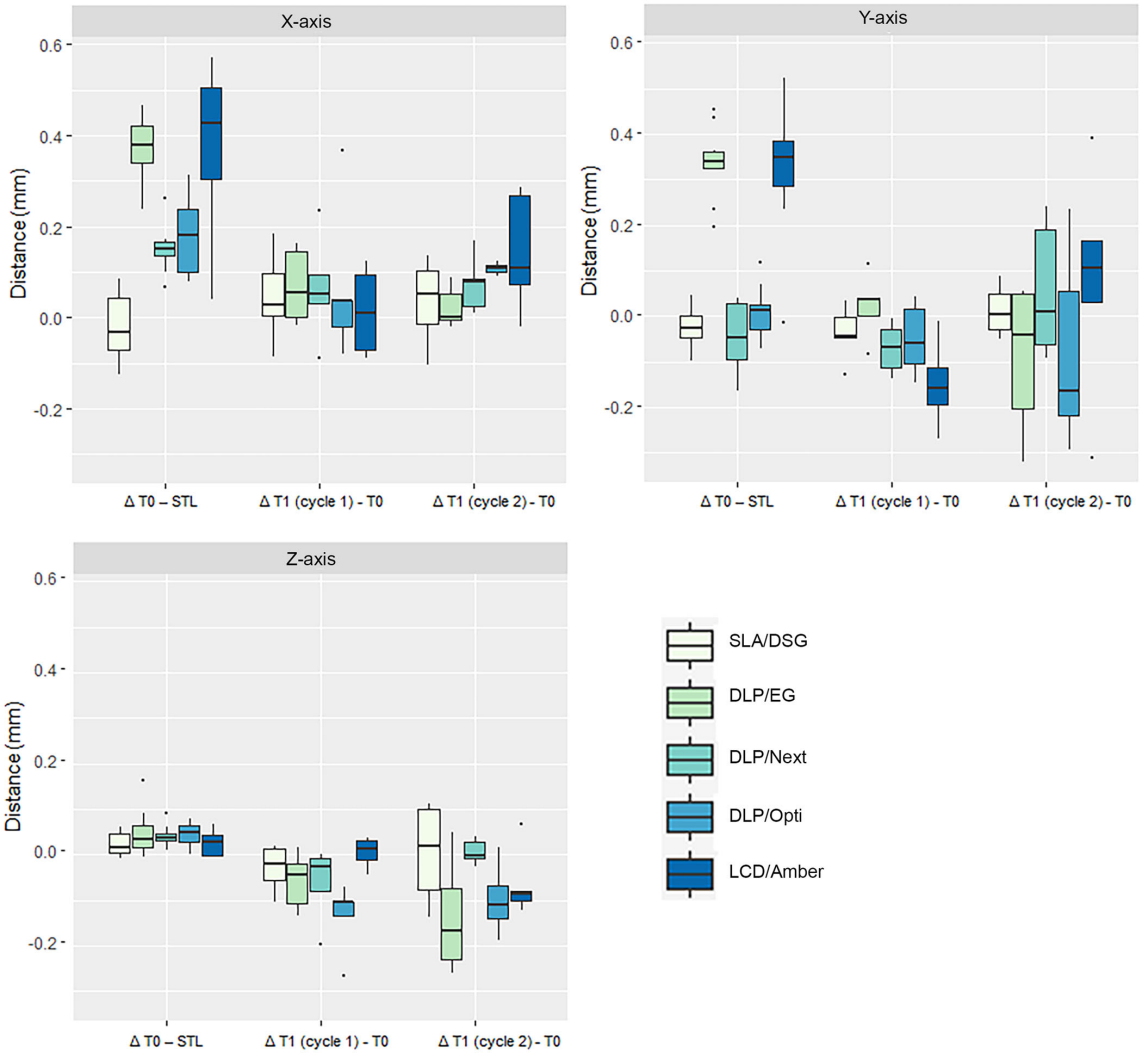
Dimensional changes in the three axes between the original.stl file (STL) and the printed guides before (T0) and after (T1) autoclaving.

Dimensional values recorded in the three axes are presented in Figure 4 and Table 2.

**Table 2:** Dimensional values (mm) in the three axes of the original STL file and of the 3D‐printed guided before (T0) and after autoclaving (T1).

Group	Axis	Original STL		T0		T1 (Cycle 1)^a^		T1 (Cycle 2)^a^		*p* values between time points/cycles
**DLP/EG**	x	39.97***^1,2,3^	[0.00]	40.35***^1^	[0.06]	40.39***^2^	[0.05]	40.39***^3^	[0.08]	< 0.001***
	y	23.07***^1,2,3^	[0.00]	23.41***^1^	[0.08]	23.43***^2^	[0.08]	23.33***^3^	[0.10]	< 0.001***
	z	5.51	[0.00]	5.56*	[0.05]	5.48	[0.08]	5.44*	[0.16]	0.026*
**DLP/Next**	x	39.97***^1,2,3^	[0.00]	40.12***^1,^*	[0.05]	40.21***^2,^*	[0.12]	40.17***^3^	[0.04]	< 0.001***
	y	23.07*	[0.00]	23.03	[0.08]	22.96*	[0.08]	23.09	[0.20]	0.048*
	z	5.51*^1,2^	[0.00]	5.55*^1,**1^	[0.02]	5.49**^1,2^	[0.06]	5.55*^2,**2^	[0.04]	< 0.001***
**DLP/Opti**	x	39.97***^1,2,3^	[0.00]	40.15***^1,^*	[0.08]	40.23***^2^	[0.18]	40.31***^3,^*	[0.17]	< 0.001***
	y	23.07	[0.00]	23.08	[0.06]	23.05	[0.08]	22.99	[0.20]	0.240
	z	5.51*	[0.00]	5.55***, **	[0.03]	5.43***, *	[0.10]	5.44**	[0.09]	< 0.001***
**SLA/DSG**	x	39.97	[0.00]	39.95	[0.07]	39.99	[0.14]	39.99	[0.10]	0.598
	y	23.07	[0.00]	23.05	[0.05]	23.01	[0.08]	23.06	[0.08]	0.098
	z	5.51	[0.00]	5.53	[0.03]	5.50	[0.04]	5.53	[0.12]	0.412
**LCD/Amber**	x	39.97***^1,2,3^	[0.00]	40.35***^1^	[0.17]	40.44***^2^	[0.15]	40.44***^3^	[0.20]	< 0.001***
	y	23.07***^1,2,3^	[0.00]	23.40***^1^	[0.15]	23.35***^2^	[0.09]	23.39***^3^	[0.15]	< 0.001***
	z	5.51	[0.00]	5.53**^1^	[0.03]	5.53**^2^	[0.01]	5.47**^1,2^	[0.06]	0.003**

*Note:* In the outer right column, *p* values from likelihood‐tests from mixed‐effects models comparing the dimensional values among time points are reported. If significant, *p* values from the post‐hoc test are coded with the following symbols: *p = < 0.05, **p = < 0.01, ***p = < 0.001.

^a^ Mean ± standard deviation in mm.

When comparing the original STL to the scans taken at T0 and T1, only SLA/DSG exhibited no significant changes in any axis. All the remaining four printer/resin combinations showed significant changes in the *x*‐axis (*p* < 0.001). Two printer/resin combinations (DLP/EG and LCD/Amber) showed significant changes in the *y*‐axis (*p* < 0.001), and DLP/Next in the *z*‐axis (*p* < 0.001).

As reported in Table 2, when comparing STL to T0, four guides presented significant expansion in the *x*‐axis, two in the *y*‐axis and only one in the *z*‐axis. The greatest changes (Δ T0 – STL) were registered in the *x*‐ and *y*‐axis, in particular in DLP/EG (*x*‐axis 0.38 mm and *y*‐axis 0.34 mm) and LCD/Amber (*x*‐axis 0.38 mm and *y*‐axis 0.33 mm), as seen in Table 2.

When comparing the scans taken at T0 to the ones at T1, DLP/Next showed significant expansion in the *x*‐axis for Cycle 1 and DLP/Opti for Cycle 2. None of the resin/printer combinations showed significant changes in the *y*‐axis when comparing T0 with T1 scans. Four out of the five investigated printer/resin combinations presented significant changes in the *z*‐axis between T0 and either Cycle 1 (DLP/Next), Cycle 2 (DLP/EG, LCD/Amber), or both (DLP/Opti).

All guides but one (SLA/DSG) presented significant expansion between STL and T1 for both cycles in the *x*‐axis. Significant differences between STL and T1 (Cycles 1 and/or 2) were detected in the *y*‐ and *z*‐axis in three (DLP/EG, DLP/Next, and LCD/Amber) and two (DLP/Next and DLP/Opti) printer/resin combinations, respectively.

When comparing Cycle 1 to Cycle 2, significant differences were found only in the *z*‐axis (DLP/Next, LDC/Amber).

## 4 Discussion

The printing process and autoclaving were found to affect the dimensional stability of four out of five 3D‐printed insertion guides. Therefore, the null‐hypothesis was rejected for all PRCs but SLA/DSG. Indeed, the Dental SG resin performed the best and interestingly it was the same material selected by other authors after preliminary tests due to the absence of dimensional imbalances and material changes after sterilization at 135°C for 18.23 min (Ludwig et al. 2022). In Ludwig et al. (2022), the sterilization process resulted in an improvement in the accuracy of CAD/CAM insertion guides for mini‐implant positioning. In particular, at the head of the implants, which is the most relevant position for the fitting of the pre‐fabricated orthodontic appliance, the lower mean deviation of the mini‐implant from the digitally planned position was observed in the sterilized group and amounted to 0.057 mm, which is considered clinically acceptable.

Several studies have demonstrated the advantages of orthodontic mini‐implant placement with the use of insertion guides over a free‐hand approach in terms of precision (Bae et al. 2013; Iodice et al. 2022). Also a reduced number of appointments may be needed if the appliance is inserted directly after implant placement, as reported by Wilmes et al. for maxillary molar distalization appliances (Wilmes et al. 2019).

Since these guides come in contact with the mucous membranes, blood and, eventually, bone, their sterilization is recommended before usage. The effect of sterilization on the insertion guides has attracted little attention from researchers so far. However, steam autoclaving is commonly accepted as being suitable for sterilizing 3D‐printed guides (Török et al. 2020). In the present study, DLP/EG presented high dimensional changes. It is to be noted that this is the only resin among the tested materials not authorized for steam autoclaving by the manufacturer. However, it was tested since it is commonly autoclaved in dental practices in the attempt to comply with the US and EU law for Medical Devices (IA/critical A) (European Parliament and Council, 5 April 2017; Rutala et al. 2008).

Although information derived from the field of surgical guides for the placement of dental implants can be partially transferred to orthodontic mini‐implant insertion guides, there is a scarcity of studies explicitly focused on the latter. In a previous study of our group, findings were reported using the same PRCs and assessment methods but with a guide design intended for dental implant insertion (Hüfner et al. 2024). In comparison with the former one, the currently investigated insertion guide presents a slenderer skeletonized rod‐like design, characterized by punctiform supporting occlusal contacts and absence of an extended structure englobing all the upper teeth with transpalatal supporting struts.

In Hüfner et al. (2024), the printing process was associated with significant dimensional changes in all the PRCs, contrary to the present study, where SLA/DSG resulted not to be affected by both the printing process and the sterilization. However, both studies registered the highest printing‐correlated expansion values in both *x*‐ and *y*‐direction using DLP/EG and LCD/Amber.

When comparing the measurements before and after autoclaving taken on the surgical guides (Hüfner et al. 2024), only the ones produced as in the DLP/EG group were not affected by the sterilization process independently of the cycle. Whereas the guides here investigated mini‐implant insertion printed with the same material and method exhibited a significant shrinkage in the *z*‐axis after Cycle 2. In detail, autoclaving‐induced changes in the *z*‐direction were noticed in all mini‐implant insertion guides but one for at least one of the cycles, while in Hüfner et al. (2024) they were registered only with DLP/Next and SLA/DSG; however in both studies, the shrinkage was below 0.1 mm. Nonetheless, it has to be noted that the study design was not composed to assess the shape of an insertion template or drilling guide has an impact on dimensional changes following printing and autoclaving. Therefore, future studies are needed to study if the template design affects dimensional changes induced by to the autoclaving and 3D‐printing process.

In a recent study, steam sterilization both at 121°C and 134°C was found to alter the mechanical properties of surgical guides for orthodontic implants and therefore the authors concluded that autoclaving is not a recommended method for the sterilization of insertion guides intended for mini‐implant placement (Pop et al. 2022).

With the rapidly expanding catalog of available printing technologies, printers, and resins (Tian et al. 2021), it has become crucial to understand how different resins printed using compatible printers perform after sterilization. In the present study, five printer/resin combinations currently utilized in clinical practice were selected and tested. The same combinations were also investigated in a previous study of our group aiming at comparing the Vickers hardness and the flexural modulus of 3D‐printed specimens specifically designed for mechanical testing (Kirschner et al. 2022). Changes in Vickers hardness were observed in 3 out of 5 groups (DLP/EG, DLP/Opti, and SLA/DSG), while a statistically significant decrease in the flexural modulus was detected only in one group (DLP/Next) after Cycle 2. Hence, using lower temperatures and pressures and longer sterilization durations might be advisable when sterilizing 3D‐printed guides (Kirschner et al. 2022).

The autoclaving‐induced dimensional changes were particularly pronounced in the *z*‐axis affecting all but one printer/resin combination (SLA/DSG) for either Cycles 1 and/or 2. However, it has to be noted that the linear measurements of the *z*‐axis were particularly difficult to be obtained as the landmark that was used to measure the *z*‐axis was set on a sloped base.

The layer height also has an impact on the accuracy when printing parallel to the print bed, as previously reported (Stansbury and Idacavage 2016). All printer/resin combinations except for DLP/EG were printed at a layer height of, similar to other studies (Oh et al. 2019; Vara et al. 2023). All investigated guides were printed parallel to the print bed with their intaglio surfaces facing away from the print bed to ensure the readability of the landmarks and to avoid printing supports interfering with the measuring process. This printing orientation was selected since a parallel placement as well as an angle of 45° to the print bed had resulted in a higher accuracy of the printed guide morphology (Dalal et al. 2020; Unkovskiy et al. 2018).

Although the printer/resin combinations behaved differently from one another, another study found clinically acceptable levels of accuracy and none of the 3D printers investigated was statistically superior (Tsolakis et al. 2022).

In the present study, only one printing orientation was chosen for all the specimens, and this may represent a limitation. Nevertheless, previous studies found this orientation to be well suited and analyzing the impact of the printing angle was not the goal of the present investigation. Due to the in vitro nature of the study, it was not possible to verify in patients the actual accuracy of orthodontic mini‐implant position with respect to the virtual planning. Furthermore, the morphology of the portion of the guide at the interface with the teeth was not evaluated and this might affect the fitting of the guide and its stability during insertion.

Finally, we investigated the effect of PRCs, since the proposed combinations are commercialized and used for clinical applications contextually. Nevertheless, the resin material itself rather than the printing process is likely to dominate the dimensional changes during autoclaving. Fourier Transform Infrared Spectroscopy (FTIR) analysis could be considered in future studies to determine possible molecular changes or degree of conversion changes through different sterilization cycles.

## 5 Conclusions

Within the scope and the limitations of this study, we can conclude that the printing process as well as the steam autoclaving could affect the dimensional stability of the 3D‐printed orthodontic mini‐implant guides. The tested printer/resin combinations behaved differently from one another and only the SLA/DSG group showed no significant changes in any of its axes. With the current skeletonized insertion guide, the deviations from the original guide design accumulated until the end of the entire workflow including autoclaving were always below 0.5 mm in all axes. Future clinical trials or ex vivo studies in human cadavers should assess the accuracy of mini‐implant placement performed with the help of rod‐like skeletonized guides and the subsequent fitting of mini‐implant anchored orthodontic appliances produced based on the digital planning. Indeed, a perfect fitting would allow the delivery of the appliance in the same appointment of mini‐implant insertion, being advantageous for both the patient and the clinician in terms of time‐saving.

## Author Contributions

**Samuel David:** methodology, software, validation, investigation, data curation, writing – original draft preparation, visualization, funding acquisition. **Mira Hüfner:** formal analysis. **Nicole Rauch:** software. **Robert Kerberger:** software, writing – review and editing. **Dieter Drescher:** resources, writing – review and editing. **Giulia Brunello:** conceptualization, writing‐original draft preparation, writing – review and editing. **Kathrin Becker:** conceptualization, methodology, formal analysis, investigation, resources, data curation, writing – review and editing, supervision, project administration, funding acquisition. All authors have read and agreed to the published version of the manuscript.

## Ethics Statement

The authors have nothing to report.

## Consent

The authors have nothing to report.

## Conflicts of Interest

The authors declare no conflicts of interest.

## Supporting information

Supporting information.

Supporting information.

## Data Availability

The data that support the findings of this study are available from the corresponding author upon reasonable request.
