# Correction to “Impact of Autoclaving on the Dimensional Stability of 3D‐Printed Guides for Orthodontic Mini‐Implant Insertion—An In Vitro Study”

**DOI:** 10.1002/cre2.70125

**Published:** 2025-04-08

David, S., Hüfner, H., Rauch, N., Kerberger, R., Drescher, D., Brunello, G., and Becker B. “Impact of Autoclaving on the Dimensional Stability of 3D‐Printed Guides for Orthodontic Mini‐Implant Insertion—An In Vitro Study.” *Clinical and Experimental Dental Research* 11, 2025: e70111.

On page 1, the text “Samuel David and Mira Hüfner contributed equally to this article.” is incorrect and should not have appeared. Samuel David is the first author and Mira Hüfner is the second author, with no shared first authorship.

We apologize for this error.

