# Supplementary material for: Impact of Autoclaving on the Dimensional Stability of 3D‐Printed Guides for Orthodontic Mini‐Implant Insertion – An In Vitro Study
Source: Clin Exp Dent Res. 2025 Mar 7;11(1):e70111. doi: 10.1002/cre2.70111 (PMC11886601; doi:10.1002/cre2.70111)
Supplement: Supplementary file 1 — Supporting information. [file CRE2-11-e70111-s002.docx]

**Appendix 1.** Modified CONSORT checklist of items for reporting in vitro studies of dental materials (from Faggion, 2012).

| **Section/Topic** | **Checklist item** | | |
| --- | --- | --- | --- |
| **Abstract** | 1 |  | Structured summary of trial design, methods, results, and conclusions |
| **Introduction** | | |  |
| Background/rationale | 2 | (a) | Scientific background and explanation of rationale |
|  |  | (b) | Specific objectives and/or hypotheses |
| **Methods** | | |  |
| Intervention | 3 |  | The intervention for each group, including how and when it was administered, with sufficient detail to enable replication |
| Outcomes | 4 |  | Completely defined, pre-specified primary and secondary measures of outcome, including how and when they were assessed |
| Sample size | 5 |  | How sample size was determined |
| Randomization:  Sequence generation | 6 |  | Method used to generate the random allocation sequence |
| Allocation concealment mechanism | 7 |  | Mechanism used to implement the random allocation sequence (for example, sequentially numbered containers), describing any steps taken to conceal the sequence until intervention was assigned |
| Implementation | 8 |  | Who generated the random allocation sequence, who enrolled teeth, and who assigned teeth to intervention |
| Blinding | 9 |  | If done, who was blinded after assignment to intervention (for example, care providers, those assessing outcomes), and how |
| Statistical method | 10 |  | Statistical methods used to compare groups for primary and secondary outcomes |
| **Results** | | |  |
| Outcomes and estimation | 11 |  | For each primary and secondary outcome, results for each group, and the estimated size of the effect and its precision (for example 95% confidence interval) |
| **Discussion** |  |  |  |
| Limitations | 12 |  | Trial limitations, addressing sources of potential bias, imprecision, and, if relevant, multiplicity of analyses |
| **Other information** | | |  |
| Funding | 13 |  | Sources of funding and other support (for example suppliers of drugs), role of funders |
| Protocol | 14 |  | Where the full trial protocol can be accessed, if available |
