# Supplementary material for: Impact of Autoclaving on the Dimensional Stability of 3D‐Printed Guides for Orthodontic Mini‐Implant Insertion – An In Vitro Study
Source: Clin Exp Dent Res. 2025 Mar 7;11(1):e70111. doi: 10.1002/cre2.70111 (PMC11886601; doi:10.1002/cre2.70111)
Supplement: Supplementary file 2 — Supporting information. [file CRE2-11-e70111-s001.docx]

**Appendix 2.** Modified CONSORT adherence.

| **Section/Topic** | **Items** | | | **Criteria adherence** | **Reference section** |
| --- | --- | --- | --- | --- | --- |
| **Abstract** | 1 |  | Yes | | Abstract |
| **Introduction** | | | |  |  |
| Background/rationale | 2 | (a) | Yes | | 1 |
|  |  | (b) | Yes | | 1 |
| **Methods** | | | |  |  |
| Intervention | 3 |  | Yes | | 2.1-2.3 |
| Outcomes | 4 |  | Yes | | 2.4-2.5 |
| Sample size | 5 |  | Yes | | 2.6 |
| Randomization:  Sequence generation | 6 |  | NA | | _ |
| Allocation concealment mechanism | 7 |  | NA | | _ |
| Implementation | 8 |  | NA | | _ |
| Blinding | 9 |  | Yes | | 2.4; 2.6 |
| Statistical method | 10 |  | Yes | | 2.7 |
| **Results** | | | |  |  |
| Outcomes and estimation | 11 |  | Yes | | 3.1-3.3 |
| **Discussion** |  |  |  | |  |
| Limitations | 12 |  | Yes | | 4 |
| **Other information** | | | |  |  |
| Funding | 13 |  | Yes | | Funding; Acknowledgments |
| Protocol | 14 |  | NA | | _ |
| **Adherence** |  |  | **11/11** | |  |

NA: not applicable
